# Supplementary material for: Key functions for the transferability of a French school-based health promotion intervention
Source: PLoS One. 2026 May 29;21(5):e0348403. doi: 10.1371/journal.pone.0348403 (PMC13221045; doi:10.1371/journal.pone.0348403)
Supplement: S1 File — (DOCX) [file pone.0348403.s001.docx]

**Interview guide for district pedagogical advisors - English translation**

| **Topics covered**  *(related indicators and criteria)* | **Questions** | | **Follow-up questions** |
| --- | --- | --- | --- |
| *Opening question* | **Can you introduce yourself in a few words?** | | |
| **Reach**  *Sollicitation*  *Motivation for principal’s participation*  *Motivation for teachers’ participation*  *Recruitment strategies*  *Motivation for district pedagogical advisors* | \| How many schools participated in the *Alliance* intervention in your district? \| \| --- \| \|  \| \| What do you think about the participation rate of schools since the start of the 2022 school year? \| \|  \| \| What do you think are the most effective strategies for including schools in a health-promoting school initiative? \| \|  \| \| Did you complete the training offered by the project team? \| \|  \| | | \| To what extent does this meet your expectations? \| \| --- \| \|  \| \| Have you encountered difficulties in getting certain schools to commit to the project?  Does the principal’s commitment have an impact on the teachers’ commitment?  Do you know why some teachers choose not to take part in the project? \| \|  \| \| Multi-partner approach  Communication \| \|  \| \| If not, why not?  After completing the training, do you think it was worthwhile? \| \|  \| |
| **Effectiveness**  *Pupils benefit from health and well-being promotion initiatives in primary school*  *Primary school pupils benefit from educational/extracurricular activities that promote health and well-being*    *Development of health promotion initiatives for primary school students*  *Changes in collective work dynamics*  *Changes in representations of key concepts*  *Development of motivation to implement health promotion actions* | \| What do you think are the positive effects and outcomes of the *Alliance* intervention that you have identified? \| \| --- \| \|  \| \| Following the implementation of the *Alliance* intervention, what positive or negative consequences surprised you? \| \|  \| \| How could we improve/reinforce the positive effects you have described? \| \|  \| | | \| At the level of the children?  At the level of the teachers?  At the level of the principals?  Have you noticed any changes in partnership work since the *Alliance* intervention?  Are you working with new partners?  How often do you interact?  In your opinion, what are the benefits of the intervention in terms of teamwork?  And in terms of innovative practices in the district?  To what extent do you think *Alliance's* support can influence teachers’ perceptions of health promotion?  In your opinion, how did the resources (educational and methodological) made available to teachers help them with the project?  How has your legitimacy in addressing the topic of health promotion evolved?  And that of the teachers?  In what ways do you feel more competent in health education? \| \| --- \| \|  \| \| What unexpected outcomes did you observe? \| \|  \| \| How can the negative effects mentioned above be limited? \| \|  \| |
| **Adoption**  *Skills required to implement the intervention*  *Appropriation of methodological resources*  *Emergence of needs and request for methodological resources*  *Reinvestment in the system* | \| What do you think of the project team's support? \| \| --- \| \|  \| \| What is your perception of training courses? \| \|  \| \| What is your opinion on the proposed methodological tools? \| \|  \| | | \| Did you feel that the support was in line with your priorities?  How would you describe the frequency of this support? \| \| --- \| \|  \| \| What do you think of their duration, frequency, and content?  Do they meet your expectations?  - Replacement  - Training time  - Topics covered and selected  - Suitable for the national education audience \| \|  \| \| Were they suited to your needs?  How did you find these tools useful?  How difficult was it to learn how to use them?  What would you change about these resources? \| \|  \| |
| **Implementation**  *Costs of the intervention (time and money)*  *Increased perceived legitimacy of acting to promote health*  *Professional climate of trust*  *Identification of barriers and facilitators across teams/parameters/subgroups*  *Comparison with the FIC reference model of implemented intervention measures* | \| What do you think are the key elements for successful training? \| \| --- \| \|  \| \| What are the strengths of the project implementation? \| \|  \| \| What are the weaknesses in the implementation of the project? \| \|  \| | | \| Were teachers replaced during this time?  Was teacher training time included in the statutory hours provided for by the national education system?  In your opinion, how did the support offered help educational staff in this health promotion initiative? \| \| --- \| \|  \| \| What could be improved?   - Institutional support - Time dedicated to continuing education - Group training and support sessions - Multidisciplinary training and work meetings where health promotion is the central theme - Topics included in school curricula - Pragmatic tools providing structured guidance on the Health Promoting Schools approach - Professional climate of trust - Identified contact person - Strengthening of the working relationship between the primary school and the town hall - Multi-partner approach - Respect for the functioning of each institution - Cross-functional coordination - External trainer \| \|  \| |
| **Maintenance**  *Commitment and conditions for sustaining the actions taken*  *Identification of internal organizations that can support the program*  *Development of a professional identity in health promotion* | \| What is the likelihood that schools will continue the actions undertaken as part of the Alliance project after this school year? \| \| --- \| \|  \| \| What do you think should be done to make other schools interested in this program? \| \|  \| | \| For what reasons?  What do you think are the facilitating factors that can support the program in the long term? \| \| --- \| \|  \| \| In your opinion, what factors enable actions to be sustained in other schools?   - Commitment of stakeholders - Communication between stakeholders - Dissemination within the educational community - Dedicated working time - Formalization of the process - Facilitating resources - Administrative recognition \| \|  \| | |
