# Supplementary material for: Key functions for the transferability of a French school-based health promotion intervention
Source: PLoS One. 2026 May 29;21(5):e0348403. doi: 10.1371/journal.pone.0348403 (PMC13221045; doi:10.1371/journal.pone.0348403)
Supplement: S2 Table — (DOCX) [file pone.0348403.s002.docx]

| **Key functions** | **Form identification** | **Theoretical forms from phase 1 (2019-2022)** | **Observed forms from phase 2 (2022-2023)*** |
| --- | --- | --- | --- |
| KF1: Institutional support from the National Education system | / | Initiation and leadership of the project by a Member of Parliament, a professor of public health, and a professor of education sciences, enabling access to the target population of primary school teachers | / |
|  | 1A | Support from the (i) National Education system (working time of National Education staff dedicated to Alliance: co-facilitation of training courses by district pedagogical advisors, designation of *Alliance* referents at the district, department, and rectorate levels facilitating contact with the research team and ensuring support at each level) **and (ii) from parliamentarians in their territories (contact with mayors, presentation of the project to elected officials).** | Support from the (i) National Education system (working time of National Education staff dedicated to Alliance: co-facilitation of training courses by district pedagogical advisors, designation of *Alliance* referents at the district, department, and rectorate levels facilitating contact with the research team and ensuring support at each level) |
|  | 1B | Support from the National Education system enabling a variety of initiatives to be implemented by teachers and after-school care staff in schools, on: lifestyle habits (e.g., sleep, diet), health skills (emotion management, conflict management, etc.), mental health, etc. | Support from the National Education system enabling a variety of initiatives to be implemented by teachers and after-school care staff in schools, on: lifestyle habits (e.g., sleep, diet), health skills (emotion management, conflict management, etc.), mental health, etc. |
|  | 1C | Inclusion of Alliance training courses in departmental training plans for teachers, as decided by rectors (with training time allocated within teachers’ continuing education) | Inclusion of Alliance training courses in departmental training plans for teachers, as decided by rectors (with training time allocated within teachers’ continuing education), **although not systematically applied: some courses were instead conducted during teachers’ personal time.** |
|  | 1D | / | **Upon rector’s decision, integration of certain schools (“cités éducatives”) in priority neighborhoods into the Alliance project** |

| **Key functions** | **Form identification** | **Theoretical forms from phase 1 (2019-2022)** | **Observed forms from phase 2 (2022-2023)*** |
| --- | --- | --- | --- |
| KF2: Interprofessional training on health promotion, aimed at teachers and after-school care staff | 2A | Departmental training of district pedagogical advisors (hierarchical trainers of teachers) by the Alliance research team, so that National Education staff can play an active role in promoting health in the area. | Departmental training of district pedagogical advisors (hierarchical trainers of teachers) by the Alliance research team **at the beginning of each school year**, so that National Education staff can play an active role in promoting health in the area. |
|  | 2B | Training of teachers carried out by the Alliance research team alone, or in collaboration with the district pedagogical advisors, or solely by the district pedagogical advisors (Cantal), and conducted in person at the schools, avoiding the need for teachers and after-school care staff to travel. | Training of teachers carried out by the Alliance research team alone (Loire, Rhône), or in collaboration with the district pedagogical advisors (Isère), or solely by the district pedagogical advisors (Cantal), and conducted in person at the schools, avoiding the need for teachers and after-school care staff to travel. |
|  | 2C | Training sessions gathering school directors, teachers, after-school care staff, district pedagogical advisors, elected officials, … | Training sessions **attended mainly** by school directors, teachers, and after-school care staff, **allowing for peer-to-peer discussions on school-related issues.**  **District pedagogical advisors and elected officials only participated in the training sessions on an occasional basis.** |
|  | 2D | Theoretical and practical input on key concepts in health promotion and its place in schools (Modules 1, 2, 3, 4, and 5) in 12 hours | Evolution of training content towards more concrete contributions on key concepts of health promotion and its place in schools (Modules 1, 2, 3, 4, and 5) **in 9 hours and implementation of facilitation techniques and use of discussion tools for the training sessions (brainstorming resources, photo expression, Régnier abacus) (Loire, Isère, Rhône) by new health promotion research officers** |

| **Key functions** | **Form identification** | **Theoretical forms from phase 1 (2019-2022)** | **Observed forms from phase 2 (2022-2023)*** |
| --- | --- | --- | --- |
| KF3: Knowledge of, and respect for, the workings of each institution, and adaptation to the needs and constraints of teachers and after-school care staff for intervention systems | 3A | Taking into account the different work schedules of the various stakeholders (joint training courses are not always possible, as teachers and cafeteria staff, for example, do not always work the same hours) and training methods that can be adapted to the schedules of teachers and after-school care staff (schedules, in-person/remote format). | Taking into account the different work schedules of the various stakeholders (joint training courses are not always possible, as teachers and cafeteria staff, for example, do not always work the same hours) and training methods that can be adapted to the schedules of teachers and after-school care staff (schedules [**evening training from 5 p.m. to 8 p.m.**], in-person/remote format [**varying training times (between 2 and 6 hours) depending on the availability of local authorities]**). |
|  | 3B | Taking into account the specific characteristics of the National Education system when contacting schools, including the delay in contact due to the school holidays and the intervention schedules (no contact with schools during the holidays or the first days of the school year); prioritizing contact with school directors during their day off and contacting schools with the hierarchy copied in. | Taking into account the specific characteristics of the national education system when contacting schools, including the delay in contact due to the school holidays and the intervention schedules (no contact with schools during the holidays or the first days of the school year, **sending reports of pupils’ distress to the school district’s representatives before the summer holidays so that they have time to deal with them)**; prioritizing contact with school directors during their day off and contacting schools with the hierarchy copied in. |
|  | 3C | During training sessions, teachers and after-school care staff choose the health promotion topics and projects to be implemented in the school according to their local context, based on the school assessment (results of student questionnaires) and their preferences. | During training sessions, teachers and after-school care staff choose the health promotion topics and projects to be implemented in the school according to their local context, based on the school assessment (results of student questionnaires) and their preferences. |
|  | 3D | Face-to-face meetings and regular exchanges with stakeholders to develop a common culture and vocabulary (training, steering committee, progress reports, meetings with specific stakeholders, etc.). | Face-to-face meetings and regular exchanges with stakeholders to develop a common culture and vocabulary (training, steering committee, progress reports, **more frequent meeting with specific stakeholders to work on the project’s future (e.g., conventions), seminars with the research team and a parliamentary assistant on the functioning of the municipalities and reflections on how to integrate them into the dynamics of the Alliance project, co-creation of communication tools)** |
|  | 3E | Identification of the distribution of tasks within the research team (1 operational project coordinator and 1 research officer dedicated to working with municipalities) by stakeholders and partners during steering committee meetings and email exchanges. | **Sharing the distribution of tasks among the new research team members (two research officers for three departments, one research officer specializing in health geography, and one qualitative research officer)** with stakeholders and partners during steering committee meetings and email exchanges, and **reassigning tasks following the maternity leave of the project’s operational coordinator.** |

| **Key functions** | **Form identification** | **Theoretical forms from phase 1 (2019-2022)** | **Observed forms from phase 2 (2022-2023)*** |
| --- | --- | --- | --- |
| KF4: Methodological support and practical resources | 4A | Support to help implement projects in schools *(support for the project approach (“démarche de projet”), provision of specific resources, assistance with action planning)* included in training hours (6 hours out of 12) | Support **tailored to the needs of schools** **(need for guidance or need for autonomy)**, included in training hours (**and extendable by 2 hours**) to help with the practical implementation of projects in schools |
|  | 4B | Provision of a set of resources (educational sessions, action sheets on sleep, screens, nutrition, etc.) for teachers and after-school care staff, and practical application during training sessions for teachers and after-school care staff on these topics. | Provision of a set of **ready-to-use, reflexive** resources (educational sessions, action sheets on sleep, screens, nutrition, etc.) for teachers and after-school care staff, and practical application during training sessions for teachers and after-school care staff on these topics. |
|  | 4C | Support staff (*health promotion research officers in Loire, Isère, and Rhône, and district pedagogical advisors in Cantal*) trained and recognized for their expertise | Support staff (*health promotion research officers in Loire, Isère, and Rhône, and district pedagogical advisors in Cantal*) trained and recognized for their expertise; **offering expert conferences on topics raised in the field (teacher well-being, physical activity in municipalities, sleep, etc.) during personal time slots.** |
|  | 4D | Use and completion of methodological tools (the “pillar sheets”) during training sessions by stakeholders, enabling the definition of the project (through the establishment of a health promotion diagnosis) and the theme of action, the development of general and operational objectives, their planning and evaluation. | **Use of the project approach (“démarche de projet”) stages, use of pupils results in the diagnostic stage, and presentation of the Edusanté label to guide teachers and staff in developing and planning their health promotion activities.** |
|  | 4E | Support based on existing actions (using the “Current situation” table to be completed by teachers and staff) and promotion of the work already carried out by teaching teams. | Support based on existing actions (using the “Current situation” table to be completed by teachers and staff) and promotion of the work already carried out by teaching teams. |

| **Key functions** | **Form identification** | **Theoretical forms from phase 1 (2019-2022)** | **Observed forms from phase 2 (2022-2023)*** |
| --- | --- | --- | --- |
| KF5: General and cross-functional project coordination | 5A | Leadership of the project’s operational coordinator, liaising between all stakeholders (politicians, funders, national education authorities, local authorities, field workers, research partners, etc.) through regular formal and informal contact | Leadership of the project’s operational coordinator, liaising between all stakeholders (politicians, funders, national education authorities, local authorities, field workers, research partners, etc.) through regular formal and informal contact **during seven months of the school year, followed by segmentation of coordination (operational and administrative) following the operational coordinator’s departure on maternity leave for five months.** |
|  | 5B | Strategic planning for the continuation of the project (funding for several positions within the research team for the duration of the project, transparency in the use of funds throughout the project, feedback to funders during steering committee meetings to justify the resources invested, fundraising, continuity of partnerships) and operational planning in conjunction with stakeholders (choosing dates for training, support, student questionnaires, etc.). | Strategic planning for the continuation of the project (funding for several positions within the research team for the duration of the project, transparency in the use of funds throughout the project, continuity of partnerships) and operational planning **by** **the two research officers** in conjunction with stakeholders (choice of dates for training, support, distribution of pupils questionnaires, etc.) |
|  | 5C | Coordinating research among the consortium’s various research laboratories and monitoring their progress | Coordinating research among the consortium’s various laboratories and monitoring their progress **by the operational coordinator for 7 months, then shared between the research officer and the scientific manager for 5 months.** |
|  | 5D | Steering committee meetings held every two months, bringing together operational and financial partners throughout the project to provide feedback (presentation of progress and results) to the Ministry of Education, justifying human resources and their commitment | Steering committee organized every two months, bringing together operational and financial partners throughout the project, allowing for feedback (presentation of progress and results) to partners justifying human and financial resources and their commitment |

| **Key functions** | **Form identification** | **Theoretical forms from phase 1 (2019-2022)** | **Observed forms from phase 2 (2022-2023)*** |
| --- | --- | --- | --- |
| KF 6: Bridging fieldwork and research to allow the intervention system to evolve over time | 6A | Data collection in schools via “pupils” and “teachers” questionnaires over 3 years (well-being at school, health literacy, psychosocial skills, perceived quality of life, etc.) | Data collection via “pupils” and “teachers” questionnaires **over 4 years** (well-being at school, health literacy, psychosocial skills, perceived quality of life, etc.) **in phase 2 schools**. |
|  | 6B | Distribution of summaries of student questionnaires completed during the last year, in order to report the results (children’s health and experiences, particularly at school) to teachers to identify issues and implement actions in schools. | Distribution of summaries of pupils questionnaires **used for diagnostic purposes** (children’s health and experiences, particularly at school) to teachers **and after-school care staff** to identify issues and implement actions in schools. |
|  | 6C | Organization of conferences led by researchers or experts on key topics for teachers and after-school care staff | Organization of conferences led by researchers or experts on key topics for teachers and after-school care staff |
|  | 6D | Co-development of training content and teaching resources by national education system staff (Alliance referents) and researchers, and creation of a position within the research team to regularly update pedagogical resources. | **Maintaining a position within the research team to regularly update pedagogical resources and adapt training courses to field expectations by specialized health promotion researchers.** |

| **Key functions** | **Form identification** | **Theoretical forms from phase 1 (2019-2022)** | **Observed forms from phase 2 (2022-2023)*** |
| --- | --- | --- | --- |
| KF 7: Clear, shared internal organization of the research team (responsible for project coordination and implementation). | 7A | For Cantal: local coordination of the project by district pedagogical advisors (organization and delivery of training and support) | For Cantal: local coordination of the project by district pedagogical advisors (organization and delivery of training and support) |
|  | 7B | For Isère, Loire, Rhône: local coordination and regular monitoring by the research team | For Isère, Loire, Rhône: local coordination and regular monitoring by the research team |
|  | 7C | Within the research team, **one position is dedicated to monitoring schools and another to monitoring municipalities.** | Within the research team, **each member ensures monitoring of both schools and municipalities in one specific area.** |
|  | 7D | Team meeting to share operational progress made by the schools and municipalities being monitored | Team meeting to share operational progress made by the schools and municipalities being monitored |
